# Changes in gut microbiota composition with age and correlations with gut inflammation in rats

**DOI:** 10.1371/journal.pone.0265430

**Published:** 2022-03-15

**Authors:** Chen Meng, Siyuan Feng, Zikai Hao, Chen Dong, Hong Liu

**Affiliations:** 1 Institute of Environmental Biology and Life Support Technology, School of Biological Science and Medical Engineering, Beihang University, Beijing, China; 2 State Key Laboratory of Software Development Environment, Beihang University, Beijing, China; 3 Laboratory of Sport Nutrition and Intelligent Cooking, Shandong Sport University, Jinan, China; 4 International Joint Research Center of Aerospace Biotechnology & Medical Engineering, Beihang University, Beijing, China; National Institute of Science Education and Research, INDIA

## Abstract

Increasing evidences indicate that gut microbiota composition is associated with multiple inflammatory diseases. However, little is known about how gut microbiota changes with age and correlations with gut inflammation at sexual maturity stage of healthy individuals. Elucidating the dynamic changes of gut microbiota in healthy individuals at the sexual maturity stage and correlations with gut inflammation can provide clues for early risk assessment of gut diseases at the sexual maturity stage. Here, the shift in gut bacteria and its relationship with gut inflammation at the sexual maturity stage were explored. Sprague–Dawley rats at the sexual maturity stage were used in this study. 16S rRNA gene sequencing was performed to decipher gut bacteria shifts from the 7th week to the 9th week, and enzyme-linked immunosorbent assay (ELISA) was used to measure gut inflammation and gut barrier permeability. We found an increase in bacterial richness with age and a decrease in bacterial diversity with age. The gut bacteria were primarily dominated by the phyla *Firmicutes* and *Bacteroides* and the genus *Prevotella*. The relative abundance of *Firmicutes* increased with age, and the relative abundance of *Bacteroides* decreased with age. There was a positive correlation between body weight and the *Firmicutes*:*Bacteroides* ratio. More and more gut microbiota participated in the host gut inflammation and barrier permeability regulation with age. *Ruminococcus* was the only gut bacteria participated in gut inflammation and barrier permeability regulation both in the 7th week and the 15th week. These results provide a better understanding of the relationship between gut bacteria and gut inflammation in sexually mature rats and show that *Ruminococcus* may be a potential indicator for early risk assessment of gut inflammation.

## Introduction

Gut bacteria are considered to be a highly complex and diverse bacterial community that contributes to body health and disease [1,2]. When gut bacteria dysbiosis occurs, it will cause various health problems. For example, inflammatory bowel disease (IBD) has been shown to be associated with gut bacteria dysbiosis [1,3–5]. Gut dysfunction leads to impaired gut barrier integrity and abnormal signals in the vagus nerve [6–8]. Impaired gut barrier integrity leads to bacterial migration, which aggravates gut inflammation and peripheral inflammation [9–11]. Gut inflammation has been shown to induce some diseases, such as depression, cardiovascular disease and Parkinson’s disease [12,13]. More than 300 million people suffer from depression, and two-thirds of them have suicidal ideation, which is the second leading cause of death among people aged 15–29 years [14].

With the development of high-throughput sequencing technology, the differences of gut bacteria between young and old people have been widely explored. For example, the dominant bacteria were *Firmicutes* in young men, while *Bacteroides* were the dominant bacteria in the elderly [15,16]. The relative abundance of class *Betaproteobacteria* was higher in the below65 age group, whereas the relative abundance of class *Gammaproteobacteria* was higher in the above65 age group [17]. Aging increases gut permeability, and translocation of lumen bacterial products has been reported [18,19]. In addition, although gut microbiota composition has been widely reported to be associated with multiple inflammatory diseases, little is known about how gut microbiota changes and the relationship with gut inflammation at the sexual maturity stage of healthy individuals. Sexual maturity is a special stage, in which the endocrine and other physiological states of the body change greatly. Elucidating the dynamic changes of gut microbiota in healthy individuals at the sexual maturity stage and its relationship with gut inflammation is critical for understanding the clues of potential biological indicators that can be used for early risk assessment of gut diseases at sexual maturity stage.

In this study, we monitored the major shifts in fecal bacteria and their relationship to gut inflammation in sexually mature rats. Humans generally become sexually mature at approximately 22–28 years old, and rats become sexually mature at approximately 6 weeks old [20,21]. Therefore, repeated fecal sampling of rats was performed from 7 weeks until 15 weeks of age under controlled conditions, which corresponds to the sexually mature stage in rats. Our results provide new insight into the correlation between gut microbiota shifts and gut inflammation in sexually mature rats.

## Materials and methods

### Animals

Male Sprague–Dawley (SD) specific pathogen-free rats (150–160 g) were obtained from Beijing SPF Biotechnology Co., Ltd. (Beijing, China). All rats were housed under a 12 h light/dark cycle at a temperature of 21–22°C and a humidity of 55 ± 5%. All rats received the same sterile standard food (SFB Biotechnology Co. Ltd., Beijing, China) and tap water. All animal procedures involved in this study were performed in strict accordance with research guidelines for the care and use of laboratory animals and were approved by the ethics committee of Beihang University.

### Experimental design

**Fig 1:**
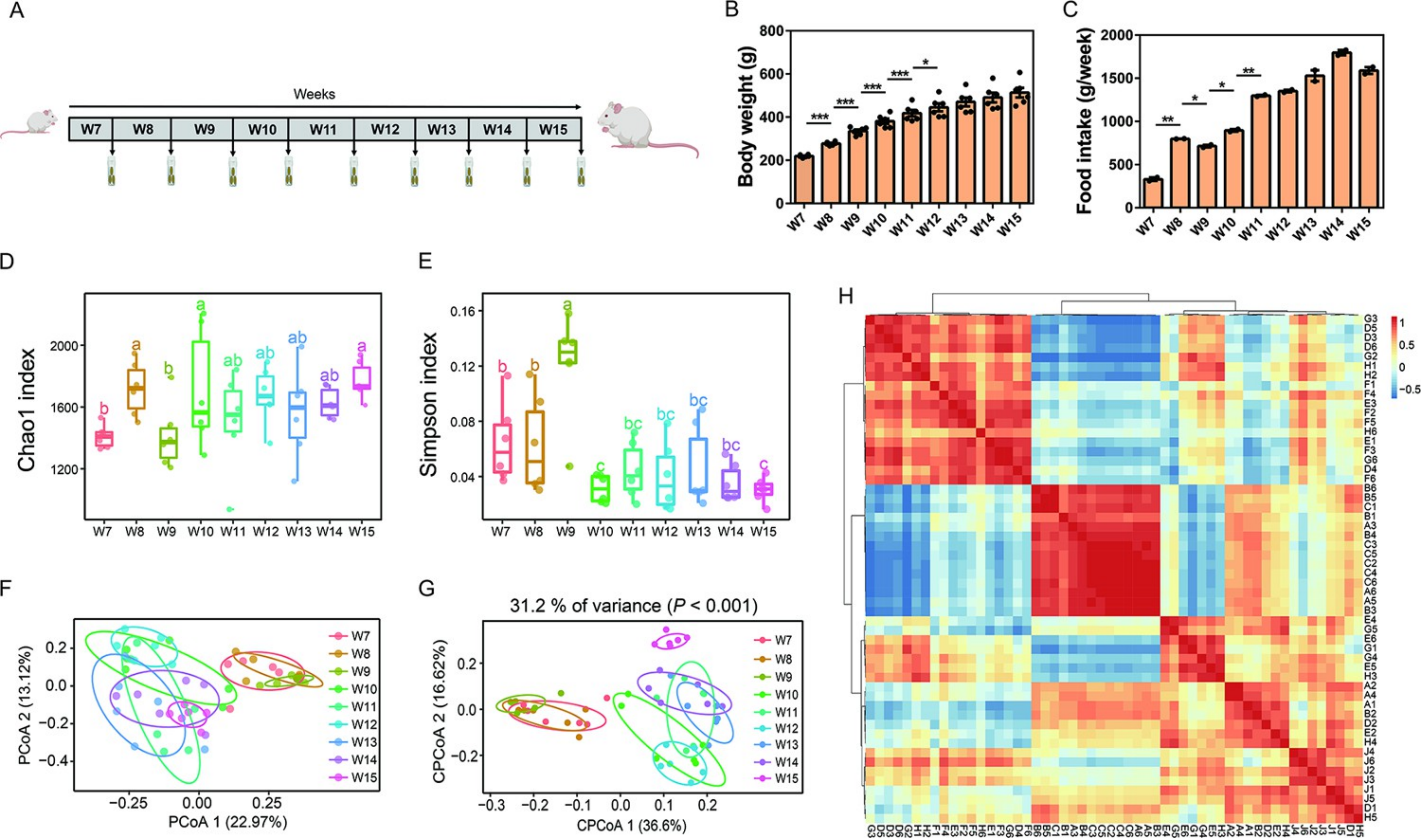
Diversity of gut bacteria shifts with time. (A), Experimental design and procedures. (B-C), Body weight and food intake shift with time (n = 6). (D-E), Chao 1 and Simpson indices were plotted to evaluate and compare α-diversity (n = 6). For boxplot with the same letter, the difference is not statistically significant. For boxplot with different letters, the difference is statistically significant. (F-G), Principal coordinate analysis and constrained principal coordinate analysis were plotted to evaluate and compare β-diversity (n = 6). (H), Hierarchical clustering using weighted UniFrac distances. OTU abundance levels were used to construct the heatmap (n = 6). Letters A-J represent the 7th week to 15th week, and numbers 1–6 represent six rats. Data were represented as means ± SEM. *P < 0.05, **P < 0.01, ***P < 0.001.

Briefly, six-week-old (n = 6) rats were allowed to adapt to the environment for one week prior to the experiment. Then, fresh feces were collected from each rat every week (9 weeks, 54 samples) and stored quickly at −80°C until analysis. The animal work presented in this study was approved by the ethics committee of Beihang University. The experimental workflow is summarized in Fig 1A.

### Information analysis of the 16S rRNA gene

#### DNA extraction and detection

At least 1 g of homogeneous fresh fecal sample was collected in a sterile sample collection tube containing phosphate buffered saline (PBS) and stored quickly at −80°C until analysis. When all samples were collected, DNA extraction was performed [22,23]. First, fecal samples were thawed on ice, and DNA was extracted by the phenol/trichloromethane/isoamyl alcohol method [24]. The extract was treated with DNase-free RNase to eliminate RNA contamination. The quality of DNA and the DNA concentration were determined by a Nanodrop spectrophotometer (Thermo Scientific, MA, USA). The molecular size of DNA was determined by agarose gel electrophoresis. According to the concentration, DNA was diluted to 1 ng/μL using sterile water.

#### Amplicon generation

16S rRNA genes (V3-V4) were amplified using 341F (5′-CCTAYGGGRBGCASCAG-3′) and 806R (5′-GGACTACNNGGGTATCTAAT-3′) primers. All PCRs were carried out with 15 μL of PCR Master Mix, 0.2 μM forward and reverse primers, and 10 ng of template DNA. Thermal cycling consisted of initial denaturation at 98°C for 1 min, followed by 30 cycles of denaturation at 98°C for 10 s, annealing at 50°C for 30 s, and elongation at 72°C for 30 s. Finally, the reaction was held at 72°C for 5 min.

#### DNA library construction and sequencing

Sequencing libraries were generated using a DNA PCR-Free Sample Preparation Kit (Illumina, CA, USA) following the manufacturer’s recommendations. The library quality was assessed on a fluorometer (Thermo Scientific, MA, USA) and Agilent Bioanalyzer 2100 system. Finally, the library was sequenced on an Illumina NovaSeq platform, and 250 bp paired-end reads were generated.

#### Microbiota analysis

The 16S rRNA gene sequences were processed using R software (Version 4.0.0, https://cran.r-project.org), USEARCH 10.0 (http://www.drive5.com/usearch/) and in-house scripts [25]. Data were visualized by using Rstudio (Version 1.3.959.0, https://rstudio.com/) and ImageGP (http://www.ehbio.com/ImageGP). Barcodes (S1 Table) and primer sequences were truncated. Sequences with ≥97% similarity were assigned to the same operational taxonomic units (OTUs). Representative sequences for each OTU were screened for further annotation. Representative sequences of OTUs were selected and analyzed with the USEARCH and Greengenes databases (Greengenes V13.5, http://www.drive5.com/sintax/gg_16s_13.5.fa.gz) to annotate taxonomic information: Kingdom, Phylum, Class, Order, Family, Genus and Species. The OTU table was standardized to the sample with the lower number of sequences and subjected to the following analyses.

Alpha diversity was used to analyze species diversity, including Chao1 and Simpson. Chao1 was selected to identify community richness, and Simpson was selected to identify community diversity. Tukey’s test and the LSD test were used to analyze differences and visualized by Rstudio (Version 1.3.959.0, https://rstudio.com/). Beta diversity was used to analyze the differences between samples in terms of species complexity and visualized by Rstudio (Version 1.3.959.0, https://rstudio.com/), including principal coordinate analysis (PCoA) analysis and constrained principal coordinate analysis (CPCoA). PCoA and CPCoA were performed to obtain principal coordinates and visualize complex, multidimensional data. A distance matrix was transformed to a new set of orthogonal axes, by which the maximum variation factor was demonstrated by the first principal coordinate, and the second maximum variation factor was demonstrated by the second principal coordinate. The vegan, ggplot2, reshape2, digest, ggrepel and ggpubr packages of R software were used in PCoA analysis. The Reshape2, ggplot2, vegan, digest, ggrepel and ggpubr packages of R software (Version 4.0.0, https://cran.r-project.org) were used in CPCoA analysis.

BugBase (https://bugbase.cs.umn.edu/) is a microbiome analysis tool that determines phenotypes present in microbiome samples. The OTU table is the input file (S2 Table) in BugBase analysis. Briefly, a standardized OTU table was input, and then the microbial phenotype was predicted by the BugBase tool. Phylogenetic Investigation of Communities by Reconstruction of Unobserved States (PICRUSt) (http://picrust.github.io/picrust/) was performed using the online version of Galaxy (http://huttenhower.sph.harvard.edu/galaxy/). 16S rRNA data in the form of a BIOM format table were selected by mapping all 16S rRNA reads to references in the Greengenes tree with OTUs assigned at 97% identity. The obtained OTU table was normalized by the 16S rRNA gene copy number, and metagenomes were predicted from the KEGG catalog.

### Cytokine measurements

Fecal samples were homogenized with phosphate-buffered saline (PBS) (60 Hz, 5 mins), and 1 g of each fecal sample was homogenized with 1 mL of PBS (1:1). White corundum was used as homogenate medium. After centrifugation at 4°C and 2800 g for 10 minutes, the fecal supernatant was extracted and stored at -80°C until analysis. A rat enzyme-linked immunosorbent kit (Beijing RGB Technology Development Co., Ltd.) was applied to measure the levels of the inflammatory cytokines TNF-α, IFN-γ, IL-6, IL-1β, corticosterone (CORT), IL-10 and lipopolysaccharide (LPS), gut barrier permeability indicators iFABP and Zonulin according to the manufacturer’s instructions. Briefly, samples were thawed, vortexed, centrifuged, incubated, and measured by ELISA (Thermo Multiskan MK3, Finland).

### Correlation analysis

Correlations between the relative abundance of individual bacteria at the genus level and cytokine expression levels were analyzed using Spearman’s rank correlation coefficient, and data were visualized by Rstudio.

### Statistical analysis

Prior to determining the significance with parametric tests, normality was tested using the D’Agostino-Pearson omnibus normality test. For normally distributed data, significance was determined using an unpaired two-tailed Student’s *t*-test. The Mann–Whitney test was used when data failed the normality test (GraphPad Software Version 6.01, Inc., La Jolla, CA, USA). Histograms and line charts are presented as the means ± standard error (SE). *P* < 0.05 was considered to indicate a statistically significant difference. Differences were noted as significant *P < 0.05, **P < 0.01, ***P < 0.001. Statistical analysis was carried out using Prism software (GraphPad Software Version 6.01, CA, USA) and OriginPro (Origin Software Version 2018C, MA, USA).

## Results

### Growth state and food intake

In this study, body weight and food intake were assessed every week. Compared with the previous week, body weight increased significantly from the 8th week to the 12th week (*P < 0*.*05*), but no significant weight gain was observed from the 13th week to the 15th week (*P* > 0.05) (Fig 1B). The average food intake increased in the 8th, 10th and 11th week, but decreased in the 9th week (Fig 1C).

### Gut bacterial diversity shifts with time

We tracked fecal samples from rats to explore gut bacterial shifts over time. A total of 2,492,337 high-quality sequences were obtained from 54 samples (ranging from 26,923 to 61,584 reads per sample). We discarded low abundance OTUs and obtained 7,242 OTUs that could be used for further analysis. Alpha diversity analysis showed that the richness (Chao1 index) of gut bacteria significantly increased with time, while the diversity (Simpson index) of gut bacteria significantly decreased with time (Fig 1D and 1E). PCoA and CPCoA were used to visualize the shifts of beta diversity with time. According to the coordinate analysis, the points of the 7th week to the 9th week were clustered together, while the points of the 10th week to the 15th week were clustered together. In addition, the 7th week to 9th week and 10th week to 15th week were obviously divided into different groups on the PC1 axis (Fig 1F and 1G), indicating that the diversity of gut bacteria shifted with time.

To identify the similarities and differences of bacterial communities at different time points, hierarchical clustering was implemented (Fig 1H). Consistent with the results of PCoA and CPCoA, two different clusters were observed between the 7th week and the 9th week and between the 10th week and the 15th week, indicating that bacterial diversity at different ages showed large shifts.

### Major shifts in the composition of gut bacteria

**Fig 2:**
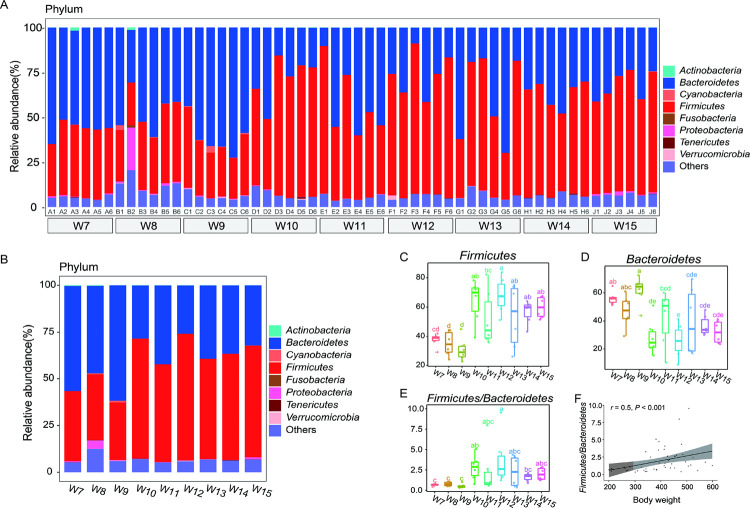
Gut bacteria composition at the phylum level. (A-B), Gut bacterial composition at the phylum level was compared for each sample and each group (n = 6). (C-E), The relative abundance of *Firmicutes* and *Bacteroides* shifted with time (n = 6), and the *Firmicutes*:*Bacteroides* ratio shifted with time (n = 6). For boxplot with the same letter, the difference is not statistically significant. For boxplot with different letters, the difference is statistically significant. (F), Spearman correlation between body weight and the *Firmicutes*:*Bacteroides* ratio (n = 54).

**Fig 3:**
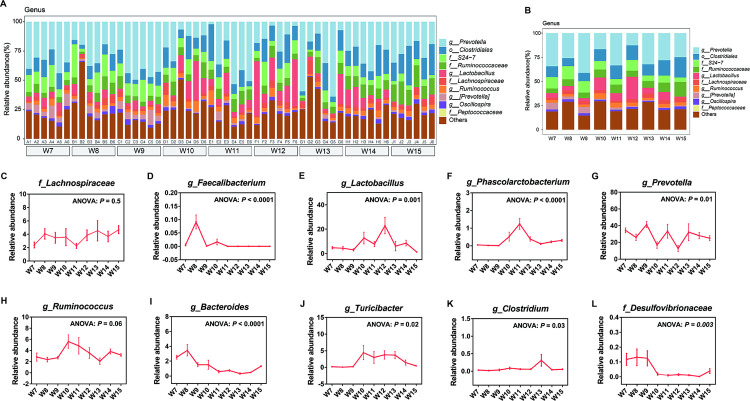
Gut bacteria composition at the genus level. (A-B), Gut bacterial composition at the genus level was compared for each sample and each group (n = 6). (C-L), Certain genera shifted with time (ANOVA test followed by post-hoc tests, n = 6).

At the phylum level, the most predominant bacterial phyla were *Firmicutes* and *Bacteroides* (Fig 2A and 2B). At the genus level, *Prevotella* was the predominant genus (Fig 3A and 3B). A significant difference was observed between the 7th week to the 9th week and between the 10th week to the 15th week at the phylum level (*P* < 0.01) (Fig 2B). The relative abundance of *Firmicutes* increased significantly from the 10th week to the 15th week, while the relative abundance of *Bacteroides* decreased significantly from the 10th week to the 15th week (Fig 2C and 2D). However, the *Firmicutes*/*Bacteroides* (*F/B*) ratio was increased from the 10th week (Fig 2E). In addition, a positive correlation between body weight and the *F/B* ratio was observed (*r* = 0.5, *P* < 0.001) (Fig 2F).

Then, we analyzed the relative abundance and shifts of potential anti-inflammatory bacteria and pro-inflammatory bacteria (Fig 3C–3L). Potential anti-inflammatory bacteria *Faecalibacterium*, *Lactobacillus*, *Phascolarctobacterium* and *Prevotella* showed obvious shift (*P* < 0.05) (Fig 3D–3L). Although potential anti-inflammatory bacteria *Lachnospiraceae* showed fluctuation, the overall fluctuation was relatively stable (*P* = 0.5). *Faecalibacterium* increased greatly in the 8th week, while *Lactobacillus* and *Phascolarctobacterium* increased greatly from the 9th week to the 13th week. Potential pro-inflammatory bacteria *Bacteroides*, *Turricibacter*, *Clostridium* and *Desulfovibrionaceae* showed a decreasing trend (*P* < 0.05), among which the relative abundance of *Bacteroides* and *Desulfovibrionaceae* showed an obvious downward trend from the 9th week to the 15th week (Fig 3I–3L).

### Gut bacteria are associated with gut inflammation

**Fig 4:**
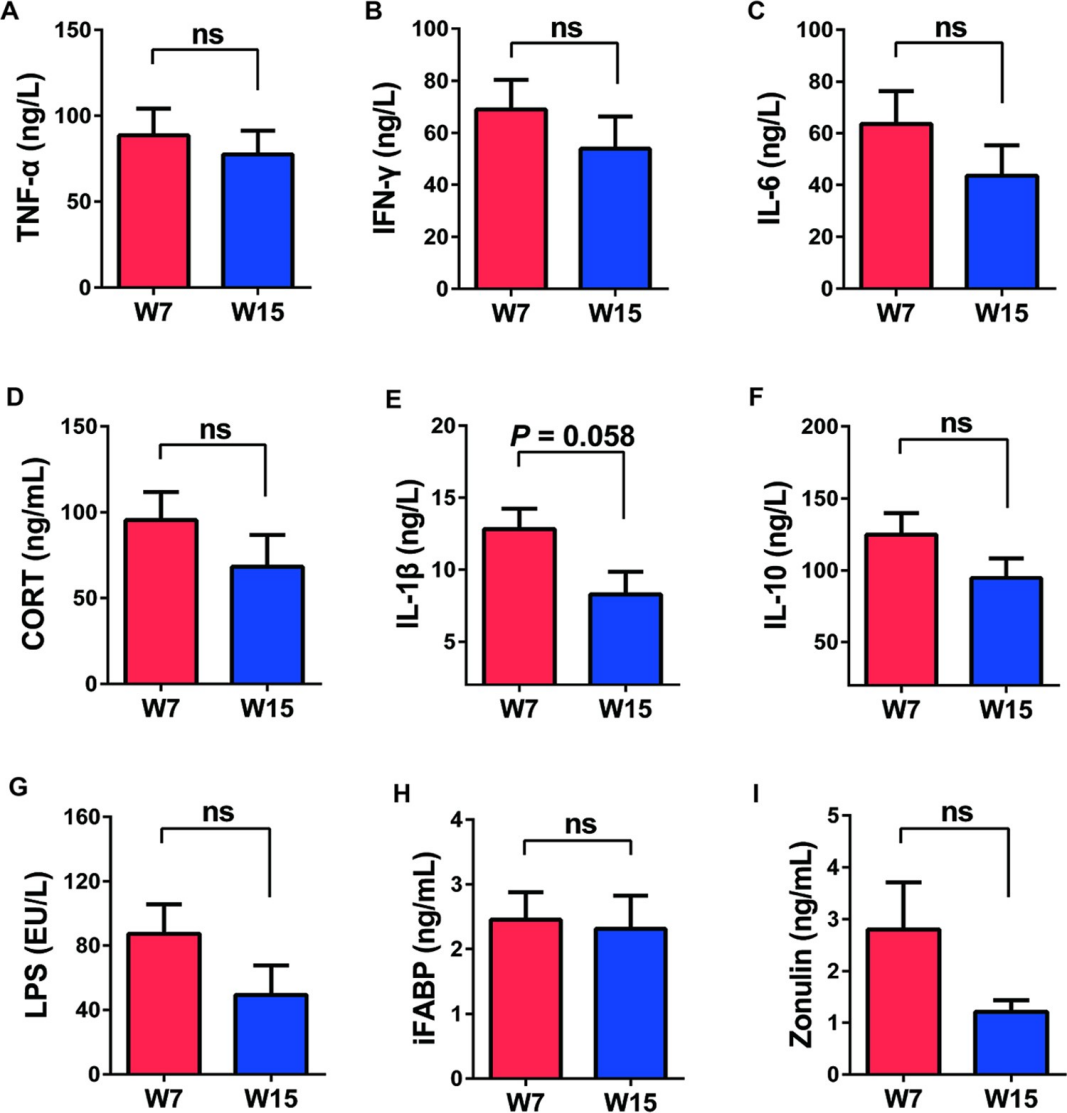
Gut inflammation, bone metabolism and gut barrier permeability-related cytokine expression in sexually mature rats. (A-G), Levels of inflammation-associated cytokines were measured in fecal samples (n = 6). (H-I), Levels of gut barrier permeability-associated cytokines were measured in fecal samples (n = 6). Data were represented as means ± SEM. ns, no significance.

As shown in Fig 4, we measured the levels of multiple cytokines (TNF-α, IFN-γ, IL-6, CORT, IL-1β, IL-10, LPS, iFABP and Zonulin). Compared with the 7th week, TNF-α, IFN-γ, IL-6, CORT, IL-1β, IL-10, LPS and Zonulin concentrations were decreased in the 15th week, but no significant differences in any of these cytokines were observed (Fig 4A–4I).

**Fig 5:**
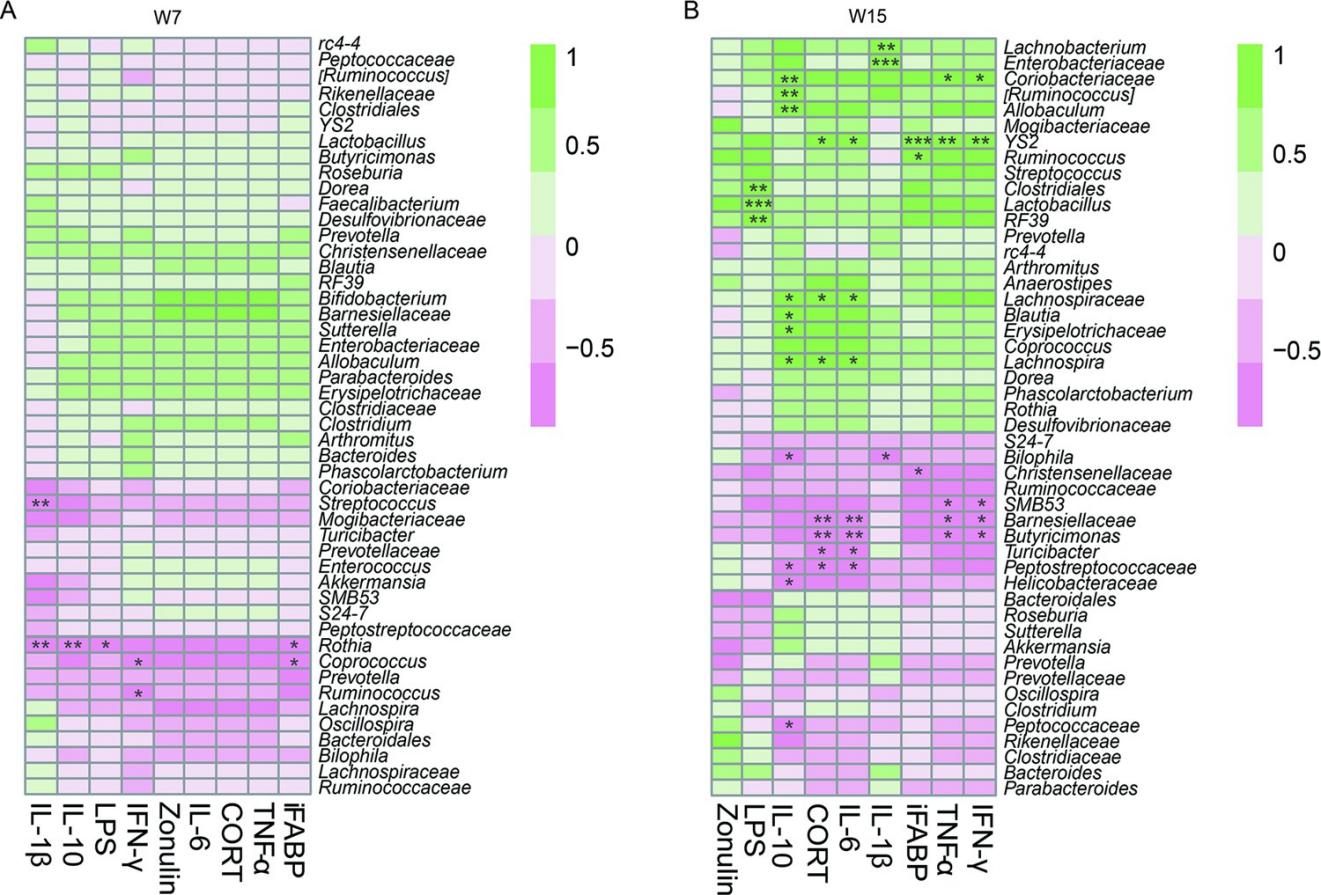
Spearman correlation between gut bacteria and gut inflammation. (A), Spearman correlation between gut bacteria and gut inflammation and gut barrier permeability-related cytokine expression in the 7th week. (B), Spearman correlation between gut bacteria and gut inflammation and gut barrier permeability-related cytokine expression in the 15th week. Note: *P < 0.05, **P < 0.01, ***P < 0.001.

Spearman correlation analysis was used to calculate the relationship between the relative abundance of gut bacteria and cytokine levels (Fig 5A and 5B). We found that *Ruminococcus* was significantly negatively correlated with IFN-γ in the 7th week (*r* = -0.8, *P* < 0.05) and significantly positively correlated with iFABP in the 15th week (*r* = 0.9, *P* < 0.05). In addition, *Clostridiales* (*r* = 0.9, *P* < 0.01) and *Lactobacillus* (*r* = 1, *P* < 0.001) were significantly positively correlated with LPS in the 15th week. *Peptococcaceae* was significantly negatively correlated with IL-10 in the 15th week (*r =* -0.8, *P* < 0.05). *Lachnospiraceae* was significantly positively correlated with CORT (*r* = 0.9, *P* < 0.05), IL-6 (*r* = 0.9, *P* < 0.05) and IL-10 (*r* = 0.8, *P* < 0.05) in the 15th week. Compared with the 7th week, the gut bacteria that correlated with gut inflammation and barrier permeability increased significantly in the 15th week, suggesting that an increasing number of gut bacteria were involved in host gut inflammation and barrier permeability regulation.

### Further insight into bacterial functions

**Fig 6:**
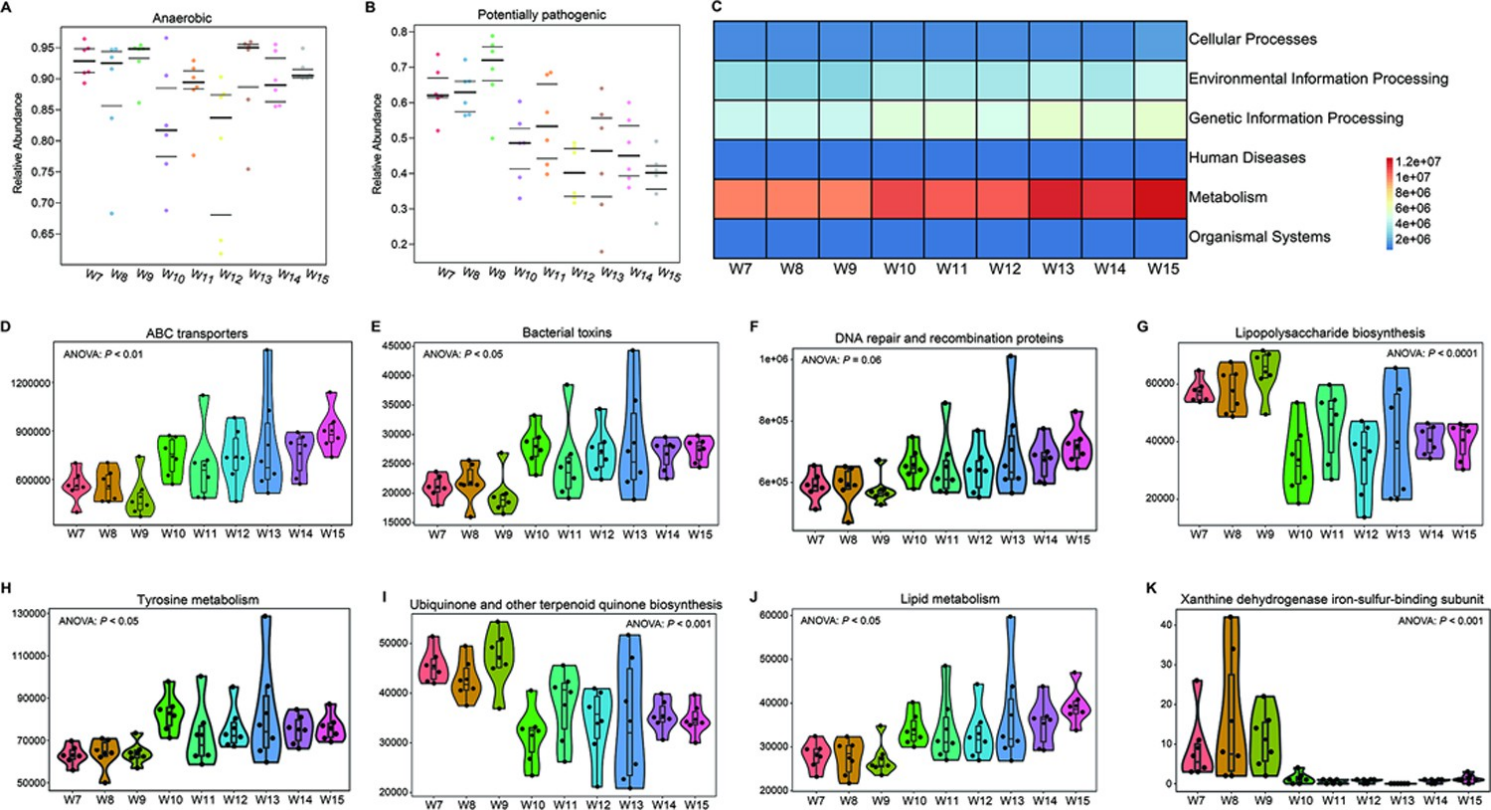
Bacterial gene function annotation and phenotype prediction. (A-B), Anaerobic and potentially pathogenic shifts with time are shown. (C-K), The average abundance of differentially enriched KEGG pathways (ANOVA test followed by post-hoc tests).

Compared with the 7th week, the relative abundance of the anaerobe phenotype showed no significant shift, and the relative abundance of the potentially pathogenic phenotype decreased with time (*P* < 0.05), suggesting that host gut bacteria were developing toward healthier states (Fig 6A and 6B).

The metabolic pathway activities of environmental information processing, genetic information processing and metabolism increased with time (Fig 6C). ABC transporters, bacterial toxins, tyrosine metabolism and lipid metabolism pathway activities were significantly increased with time (*P* < 0.05) (Fig 6D, 6E, 6H and 6J). DNA repair and recombinant protein pathway activities showed an increased trend (*P* = 0.06) (Fig 6F). The activities of lipopolysaccharide biosynthesis, ubiquinone and other terpenoid quinone biosynthesis, and xanthine dehydrogenase iron-sulfur-binding subunit metabolic pathway activities were significantly decreased with time (*P* < 0.001) (Fig 6G, 6I and 6K). Meanwhile, there was an obvious distinction in metabolic pathway activity between the 7th week and the 9th week and between the 10th week and the 15th week (Fig 6D–6K).

## Discussion

Gut bacterial development is a crucial factor affecting host inflammation, immunity and disease [26,27]. However, a study on the functional characterization of gut bacterial strains has not been conducted, and the role of bacteria in different diseases is not completely consistent. We found that the gut bacterial community shifted in a successional manner, and the bacterial community developed with increasing richness and decreasing diversity with age.

*Lachnospiraceae* was decreased in children with diarrhea, and supplementation with *Lachnospiraceae* alleviated obesity and inflammation in mice [28,29]. In this study, we found that *Lachnospiraceae* was positively associated with the levels of the anti-inflammatory cytokine IL-10, suggesting that *Lachnospiraceae* may play positive roles in host gut inflammation. *Ruminococcus* was negatively associated with pro-inflammatory cytokine IFN-γ levels and positively associated with iFABP, suggesting that *Ruminococcus* may also play positive roles in host gut inflammation. We found that *Lactobacillus* was strongly positively associated with LPS levels, an endotoxin that promotes the synthesis and release of various cytokines and inflammatory mediators. Greatly increases in *Lactobacillus* abundance from the 9th week to 13th week may imply that the body suffers from some abnormal changes and more *Lactobacillus* were needed. In addition, *Lachnospiraceae*, *Faecalibacterium*, *Ruminococcus* and *Lactobacillus* were considered to be related to the expression of short-chain fatty acids (SCFAs) [30–33]. Studies have suggested that SCFAs have an important role in host metabolism, such as improving poststroke recovery, reducing the development of severe pancreatic insulitis, alleviating psychological problems and influencing the gut-brain axis [34–39]. *Bacteroides*, *Desulfovibrionaceae* and *Turricibacter* were considered to have potential proinflammatory properties in some studies [40–44]. However, we found that *Turricibacter* was significantly negatively correlated with the proinflammatory cytokine IL-6, suggesting that *Turricibacter* may have potential anti-inflammatory properties. There was a decreasing trend of gut inflammation over the sexual maturity stage in this study, which was consistent with the trend of LPS biosynthesis pathway activity in functional prediction.

In addition, it is worth noting that the results of this study were based on male rats. There is always controversy about whether male or female rats should be used in the experiment. Both female and male rats are affected by hormones, but males change more in one day, while females change more in the long term and have more variables [45]. Females and males change through different ways and different time scales. It is worth noting that the differences in physiological structure, hormone secretion level and secretory cycle between female and male rats may lead to the differences in research results.

In summary, we tracked the changes in gut bacteria and gut inflammation with growth. We found a gradual increase in bacterial richness with age and a gradual decrease in bacterial diversity with age. More and more gut microbiota participated in the host gut inflammation and barrier permeability regulation with age, and *Ruminococcus* was the only gut bacteria participated in gut inflammation and barrier permeability regulation both in the 7th week and the 15th week in this study. These results provide a better understanding of the relationship between gut bacteria and gut inflammation with age in sexually mature rats.

## Ethical approval

All procedures were performed according to the National Institutes of Health Guide for the care and use of laboratory animals, rats were euthanized with carbon dioxide and were approved by the ethics committee of Beihang University.

## Supporting information

S1 Table Barcodes and primer sequences information. (XLSX)

S2 Table The normalized OTUs in BugBase analysis. (XLSX)

## References

[1] Fung TC, Olson CA, Hsiao EY. Interactions between the microbiota, immune and nervous systems in health and disease. Nat Neurosci. 2017;20(2):145–55. Epub 2017/01/16. doi: 10.1038/nn.4476 ; PubMed Central PMCID: PMC6960010. 28092661 PMC6960010

[2] Cryan JF, O’Riordan KJ, Cowan CSM, Sandhu KV, Bastiaanssen TFS, Boehme M, et al. The microbiota-gut-brain axis. Physiol Rev. 2019;99(4):1877–2013. Epub 2019/08/29. doi: 10.1152/physrev.00018.2018 . 31460832

[3] Soto M, Herzog C, Pacheco JA, Fujisaka S, Bullock K, Clish CB, et al. Gut microbiota modulate neurobehavior through changes in brain insulin sensitivity and metabolism. Mol Psychiatry. 2018;23(12):2287–301. Epub 2018/06/18. doi: 10.1038/s41380-018-0086-5 ; PubMed Central PMCID: PMC6294739. 29910467 PMC6294739

[4] Caspani G, Kennedy S, Foster JA, Swann J. Gut microbial metabolites in depression: understanding the biochemical mechanisms. Microb Cell. 2019;6(10):454–81. Epub 2019/09/27. doi: 10.15698/mic2019.10.693 ; PubMed Central PMCID: PMC6780009. 31646148 PMC6780009

[5] Sheehan D, Shanahan F. The gut microbiota in inflammatory bowel disease. Gastroenterol Clin North Am. 2017;46(1):143–54. Epub 2017/02/07. doi: 10.1016/j.gtc.2016.09.011 . 28164847

[6] Bonaz B, Sinniger V, Pellissier S. Anti-inflammatory properties of the vagus nerve: potential therapeutic implications of vagus nerve stimulation. J Physiol. 2016;594(20):5781–90. Epub 2016/04/10. doi: 10.1113/JP271539 ; PubMed Central PMCID: PMC5063949. 27059884 PMC5063949

[7] Bercik P, Verdu EF, Foster JA, Macri J, Potter M, Huang X, et al. Chronic gastrointestinal inflammation induces anxiety-like behavior and alters central nervous system biochemistry in mice. Gastroenterology. 2010;139(6):2102–12. Epub 2010/06/27. doi: 10.1053/j.gastro.2010.06.063 . 20600016

[8] Bellavance MA, Rivest S. The HPA-immune axis and the immunomodulatory actions of glucocorticoids in the brain. Front Immunol. 2014;5:136. Epub 2014/04/20. doi: 10.3389/fimmu.2014.00136 ; PubMed Central PMCID: PMC3978367. 24744759 PMC3978367

[9] Stevens BR, Goel R, Seungbum K, Richards EM, Holbert RC, Pepine CJ, et al. Increased human intestinal barrier permeability plasma biomarkers zonulin and FABP2 correlated with plasma LPS and altered gut microbiome in anxiety or depression. Gut. 2018;67(8):1555–7. Epub 2017/08/16. doi: 10.1136/gutjnl-2017-314759 ; PubMed Central PMCID: PMC5851874. 28814485 PMC5851874

[10] Rogers GB, Keating DJ, Young RL, Wong ML, Licinio J, Wesselingh S. From gut dysbiosis to altered brain function and mental illness: mechanisms and pathways. Mol Psychiatry. 2016;21(6):738–48. Epub 2016/04/19. doi: 10.1038/mp.2016.50 ; PubMed Central PMCID: PMC4879184. 27090305 PMC4879184

[11] Kelly JR, Kennedy PJ, Cryan JF, Dinan TG, Clarke G, Hyland NP. Breaking down the barriers: the gut microbiome, intestinal permeability and stress-related psychiatric disorders. Front Cell Neurosci. 2015;9:392. Epub 2015/10/14. doi: 10.3389/fncel.2015.00392 ; PubMed Central PMCID: PMC4604320. 26528128 PMC4604320

[12] Newcomer JW, Hennekens CH. Severe mental illness and risk of cardiovascular disease. JAMA. 2007;298(15):1794–6. Epub 2007/10/17. doi: 10.1001/jama.298.15.1794 . 17940236

[13] Ishihara L, Brayne C. A systematic review of depression and mental illness preceding Parkinson’s disease. Acta Neurol Scand. 2006;113(4):211–20. Epub 2006/03/18. doi: 10.1111/j.1600-0404.2006.00579.x . 16542159

[14] Malhi GS, Mann JJ. Depression. Lancet. 2018;392(10161):2299–312. Epub 2018/11/07. doi: 10.1016/S0140-6736(18)31948-2 . 30396512

[15] Claesson MJ, Cusack S, O’Sullivan O, Greene-Diniz R, Weerd HD, Flannery E, et al. Composition, variability, and temporal stability of the intestinal microbiota of the elderly. Proc Natl Acad Sci USA. 2011;108 (Suppl 1):4586–91. Epub 2010/06/24. doi: 10.1073/pnas.1000097107 ; PubMed Central PMCID: PMC3063589. 20571116 PMC3063589

[16] Takagi T, Naito Y, Inoue R, Kashiwagi S, Uchiyama K, Mizushima K, et al. Differences in gut microbiota associated with age, sex, and stool consistency in healthy Japanese subjects. J Gastroenterol. 2019;54(1):53–63. Epub 2018/06/22. doi: 10.1007/s00535-018-1488-5 . 29926167

[17] Herzog EL, Wäfler M, Keller I, Wolf S, Zinkernagel MS, Zysset-Burri DC. The importance of age in compositional and functional profiling of the human intestinal microbiome. PLoS One. 2021;16(10):e0258505. Epub 2021/10/19. doi: 10.1371/journal.pone.0258505 ; PubMed Central PMCID: PMC8523055. 34662347 PMC8523055

[18] Qi Y, Goel R, Kim S, Richards EM, Carter CS, Pepine CJ, et al. Intestinal permeability biomarker zonulin is elevated in healthy aging. J Am Med Dir Assoc. 2017;18(9):810.e1-.e4. Epub 2017/07/06. doi: 10.1016/j.jamda.2017.05.018 ; PubMed Central PMCID: PMC5581307. 28676292 PMC5581307

[19] Guigoz Y, Doré J, Schiffrin EJ. The inflammatory status of old age can be nurtured from the intestinal environment. Curr Opin Clin Nutr Metab Care. 2008;11(1):13–20. Epub 2007/12/20. doi: 10.1097/MCO.0b013e3282f2bfdf . 18090652

[20] Andreollo NA, Santos EF, Araújo MR, Lopes LR. Rat’s age versus human’s age: what is the relationship? Arq Bras Cir Dig. 2012;25(1):49–51. Epub 2012/05/10. doi: 10.1590/s0102-67202012000100011 22569979

[21] Quinn R. Comparing rat’s to human’s age: how old is my rat in people years? Nutrition. 2005;21(6):775–7. Epub 2005/06/01. doi: 10.1016/j.nut.2005.04.002 . 15925305

[22] Gorzelak MA, Gill SK, Tasnim N, Ahmadi-Vand Z, Jay M, Gibson DL. Methods for improving human gut microbiome data by reducing variability through sample processing and storage of stool. PLoS One. 2015;10(8):e0134802. Epub 2015/08/08. doi: 10.1371/journal.pone.0134802 ; PubMed Central PMCID: PMC4529225. 26252519 PMC4529225

[23] Song SJ, Amir A, Metcalf JL, Amato KR, Xu ZZ, Humphrey G, et al. Preservation methods differ in fecal microbiome stability, affecting suitability for field studies. mSystems. 2016;1(3):e00021–16. Epub 2016/11/09. doi: 10.1128/mSystems.00021-16 ; PubMed Central PMCID: PMC5069758. 27822526 PMC5069758

[24] Kumar J, Kumar M, Gupta S, Ahmed V, Bhambi M, Pandey R, et al. An improved methodology to overcome key issues in human fecal metagenomic DNA extraction. Genomics Proteomics Bioinformatics. 2016;14(6):371–8. Epub 2016/11/27. doi: 10.1016/j.gpb.2016.06.002 ; PubMed Central PMCID: PMC5200916. 27888152 PMC5200916

[25] Zhang J, Zhang N, Liu YX, Zhang X, Hu B, Qin Y, et al. Root microbiota shift in rice correlates with resident time in the field and developmental stage. Sci China Life Sci. 2018;61(6):613–21. Epub 2018/03/28. doi: 10.1007/s11427-018-9284-4 . 29582350

[26] Rooks MG, Garrett WS. Gut microbiota, metabolites and host immunity. Nat Rev Immunol. 2016;16(6):341–52. Epub 2016/05/28. doi: 10.1038/nri.2016.42 ; PubMed Central PMCID: PMC5541232. 27231050 PMC5541232

[27] Vallescolomer M, Falony G, Darzi Y, Tigchelaar EF, Wang J, Tito RY, et al. The neuroactive potential of the human gut microbiota in quality of life and depression. Nat Microbiol. 2019;4(4):623–32. Epub 2019/02/04. doi: 10.1038/s41564-018-0337-x . 30718848

[28] Castro-Mejía JL, O’Ferrall S, Krych Ł, O’Mahony E, Namusoke H, Lanyero B, et al. Restitution of gut microbiota in ugandan children administered with probiotics (*Lactobacillus rhamnosus* GG and *Bifidobacterium animalis* subsp. *lactis* BB-12) during treatment for severe acute malnutrition. Gut Microbes. 2020;11(4):855–67. Epub 2020/01/22. doi: 10.1080/19490976.2020.1712982 ; PubMed Central PMCID: PMC7524335. 31959047 PMC7524335

[29] Truax AD, Chen L, Tam JW, Cheng N, Guo H, Koblansky AA, et al. The inhibitory innate immune sensor NLRP12 maintains a threshold against obesity by regulating gut microbiota homeostasis. Cell Host Microbe. 2018;24(3):364–78. Epub 2018/09/14. doi: 10.1016/j.chom.2018.08.009 ; PubMed Central PMCID: PMC6161752. 30212649 PMC6161752

[30] Ze X, Duncan SH, Louis P, Flint HJ. *Ruminococcus bromii* is a keystone species for the degradation of resistant starch in the human colon. ISME J. 2012;6(8):1535–43. Epub 2012/02/22. doi: 10.1038/ismej.2012.4 ; PubMed Central PMCID: PMC3400402. 22343308 PMC3400402

[31] Reichardt N, Duncan SH, Young P, Belenguer A, Leitch CM, Scott KP, et al. Phylogenetic distribution of three pathways for propionate production within the human gut microbiota. ISME J. 2014;8(6):1323–35. Epub 2014/02/21. doi: 10.1038/ismej.2014.14 ; PubMed Central PMCID: PMC4030238. 24553467 PMC4030238

[32] Barcenilla A, Pryde SE, Martin JC, Duncan SH, Stewart CS, Henderson C, et al. Phylogenetic relationships of butyrate-producing bacteria from the human gut. Appl Environ Microbiol. 2000;66(4):1654–61. Epub 2000/04/01. doi: 10.1128/AEM.66.4.1654-1661.2000 ; PubMed Central PMCID: PMC92037. 10742256 PMC92037

[33] Bravo JA, Forsythe P, Chew MV, Escaravage E, Savignac HM, Dinan TG, et al. Ingestion of *Lactobacillus* strain regulates emotional behavior and central GABA receptor expression in a mouse via the vagus nerve. Proc Natl Acad Sci USA. 2011;108(38):16050–5. Epub 2011/08/31. doi: 10.1073/pnas.1102999108 ; PubMed Central PMCID: PMC3179073. 21876150 PMC3179073

[34] Sadler R, Cramer JV, Heindl S, Kostidis S, Betz D, Zuurbier KR, et al. Short-chain fatty acids improve poststroke recovery via immunological mechanisms. J Neurosci. 2020;40(5):1162–73. Epub 2020/01/01. doi: 10.1523/JNEUROSCI.1359-19.2019 ; PubMed Central PMCID: PMC6989004. 31889008 PMC6989004

[35] Xiao L, Land BV, Engen PA, Naqib A, Green SJ, Nato A, et al. Human milk oligosaccharides protect against the development of autoimmune diabetes in NOD-mice. Sci Rep. 2018;8(1):3829. Epub 2018/03/03. doi: 10.1038/s41598-018-22052-y ; PubMed Central PMCID: PMC5832804. 29497108 PMC5832804

[36] Puddu A, Sanguineti R, Montecucco F, Viviani GL. Evidence for the gut microbiota short-chain fatty acids as key pathophysiological molecules improving diabetes. Mediators Inflamm. 2014;2014:162021. Epub 2014/09/13. doi: 10.1155/2014/162021 ; PubMed Central PMCID: PMC4151858. 25214711 PMC4151858

[37] Lachmandas E, van den Heuvel CN, Damen MS, Cleophas MC, Netea MG, Crevel RV. Diabetes mellitus and increased tuberculosis susceptibility: the role of short-chain fatty acids. J Diabetes Res. 2016;2016:6014631. Epub 2016/04/09. doi: 10.1155/2016/6014631 ; PubMed Central PMCID: PMC4709651. 27057552 PMC4709651

[38] Sampson TR, Mazmanian SK. Control of brain development, function, and behavior by the microbiome. Cell Host Microbe. 2015;17(5):565–76. Epub 2015/05/15. doi: 10.1016/j.chom.2015.04.011 ; PubMed Central PMCID: PMC4442490. 25974299 PMC4442490

[39] Dalile B, Oudenhove LV, Vervliet B, Verbeke K. The role of short-chain fatty acids in microbiota-gut-brain communication. Nat Rev Gastroenterol Hepatol. 2019;16(8):461–78. Epub 2019/05/28. doi: 10.1038/s41575-019-0157-3 . 31123355

[40] Ryan FJ, Ahern AM, Fitzgerald RS, Laserna-Mendieta EJ, Power EM, Clooney AG, et al. Colonic microbiota is associated with inflammation and host epigenomic alterations in inflammatory bowel disease. Nat Commun. 2020;11(1):1512. Epub 2020/04/07. doi: 10.1038/s41467-020-15342-5 ; PubMed Central PMCID: PMC7089947. 32251296 PMC7089947

[41] Lindberg AA, Weintraub A, Zähringer U, Rietschel ET. Structure-activity relationships in lipopolysaccharides of Bacteroides fragilis. Rev Infect Dis. 1990;12:S133–S41. Epub 1990/01/01. doi: 10.1093/clinids/12.supplement_2.s133 2406867

[42] Zhang C, Zhang M, Wang S, Han R, Cao Y, Hua W, et al. Interactions between gut microbiota, host genetics and diet relevant to development of metabolic syndromes in mice. ISME J. 2010;4(2):232–41. Epub 2009/10/30. doi: 10.1038/ismej.2009.112 . 19865183

[43] Gomez A, Luckey D, Yeoman CJ, Marietta EV, Miller MEB, Murray JA, et al. Loss of sex and age driven differences in the gut microbiome characterize arthritis-susceptible 0401 mice but not arthritis-resistant 0402 mice. PLoS One. 2012;7(4):e36095. Epub 2012/05/04. doi: 10.1371/journal.pone.0036095 ; PubMed Central PMCID: PMC3338357. 22553482 PMC3338357

[44] Munyaka PM, Rabbi MF, Khafipour E, Ghia JE. Acute dextran sulfate sodium (DSS)-induced colitis promotes gut microbial dysbiosis in mice. J Basic Microbiol. 2016;56(9):986–98. Epub 2016/04/27. doi: 10.1002/jobm.201500726 . 27112251

[45] Smarr BL, Grant AD, Zucker I, Prendergast BJ, Kriegsfeld LJ. Sex differences in variability across timescales in BALB/c mice. Biol Sex Differ. 2017;8:7. Epub 2017/02/17. doi: 10.1186/s13293-016-0125-3 ; PubMed Central PMCID: PMC5301430. 28203366 PMC5301430

